# Self Capacitance Mismatch Calibration Technique for Fully-Differential Touch Screen Panel Self Capacitance Sensing System

**DOI:** 10.3390/s23073779

**Published:** 2023-04-06

**Authors:** Siheon Seong, Sewon Lee, Sunghyun Bae, Minjae Lee

**Affiliations:** School of Electrical Engineering and Computer Science, Gwangju Institute of Science and Technology, Gwangju 61005, Republic of Korea; siheonseong@gist.ac.kr (S.S.); seonelee@gist.ac.kr (S.L.); shbae@gist.ac.kr (S.B.)

**Keywords:** analog front-end (AFE), touch screen panel (TSP), self-capacitance sensing

## Abstract

This paper presents a fully-differential touch screen panel (TSP) self-capacitance sensing (SCS) system with a self-capacitance mismatch calibration technique. Due to the self-capacitance mismatch of TSP, the analog front-end (AFE) of the receiver (RX) circuit suffers from dynamic range degradation and gain limitations, which lead to the signal-to-noise ratio (SNR) loss for the TSP SCS system. The proposed calibration introduces the difference in input resistance and the driving amplifier’s strength between the fully-differential input. Thus, the mismatch effect is efficiently relieved in terms of area and power consumption. The proposed calibration restores the SNR by 19.54 dB even under the worst self-capacitance mismatch case.

## 1. Introduction

Capacitive touch screen panels (TSP) have been widely used for mobile devices. There are two types of capacitive touch sensing; mutual capacitance sensing (MCS) and self-capacitance sensing (SCS) [1]. Since MCS and SCS react differently to touch or water droplets, SCS has been utilized to correctly distinguish the actual touch and water droplets on display [2,3].

To provide a better form factor for mobile devices, TSP has become thinner these days. As a result, the base self-capacitance of organic light emitting diode (OLED) TSP increased by up to several hundred pF [4,5], and it becomes a design challenge for the SCS system. For the SCS system adopting a single-ended receiver (RX) circuit, the base capacitance occupies a large portion of the dynamic range of the analog front-end (AFE). Therefore, it requires offset compensation circuits to remove the effect of the base self-capacitance and maximize the gain for sensing the self-capacitance variation by touch [5]. Compared to the single-ended SCS system, the fully-differential SCS system [6], or the charge-sharing-based SCS system [7,8] naturally removes the offset signal because they sense the self-capacitance difference between two adjacent TSP electrodes. However, because of the self-capacitance mismatch between electrode channels, both the fully-differential- and charge-sharing-based SCS systems still suffer from dynamic range degradation.

We can briefly evaluate this sensing difficulty that arises from the large base self-capacitance and its mismatch with the electrical parameters for our design target TSP, considering specific details are withheld due to the confidentiality of the TSP manufacturer. For the RX electrodes, its base self-capacitance, $C_{p}$, is approximately 280 pF, while its variation by touch, $\Delta C_{p}$, is only about 40 fF. In other words, the single-ended SCS system should be able to sense around 0.014% variation of $C_{p}$. For the fully-differential SCS system, this large base $C_{p}$ is removed naturally. However, the self-capacitance mismatch should be considered, which is caused by the TSP fabrication mismatch and the curvature on the display edge. For the RX electrode of our design target TSP, this $C_{p}$ mismatch can be up to 12%, resulting in a maximum 33.6 pF $C_{p}$ difference. This mismatch is still 840 times larger than $\Delta C_{p}$, even after much of the base $C_{p}$ is removed by the fully-differential operation. Thus, the self-capacitance mismatch could limit the touch system signal-to-noise ratio (SNR) or, even worse, saturate the AFE and make the SCS system unable to detect touch action.

We present the self-capacitance mismatch calibration technique for the fully-differential SCS system. By adjusting the driving strength and input resistance for each fully-differential input, the proposed system reduces the RX AFE output offset induced by the self-capacitance mismatch. Therefore, the SNR of the proposed TSP SCS system can be restored without consuming excessive power and die area.

The remainder of this paper is organized as follows: Section 2 explains the output offset generation mechanism due to the self-capacitance mismatch. Section 3 presents the proposed self-capacitance calibration. Section 4 describes the implementation of the proposed TSP SCS system, including the proposed calibration. Section 5 presents the results. Section 6 concludes this paper.

## 2. Output Offset Generation Due to the Self-Capacitance Mismatch

**Figure 1:**
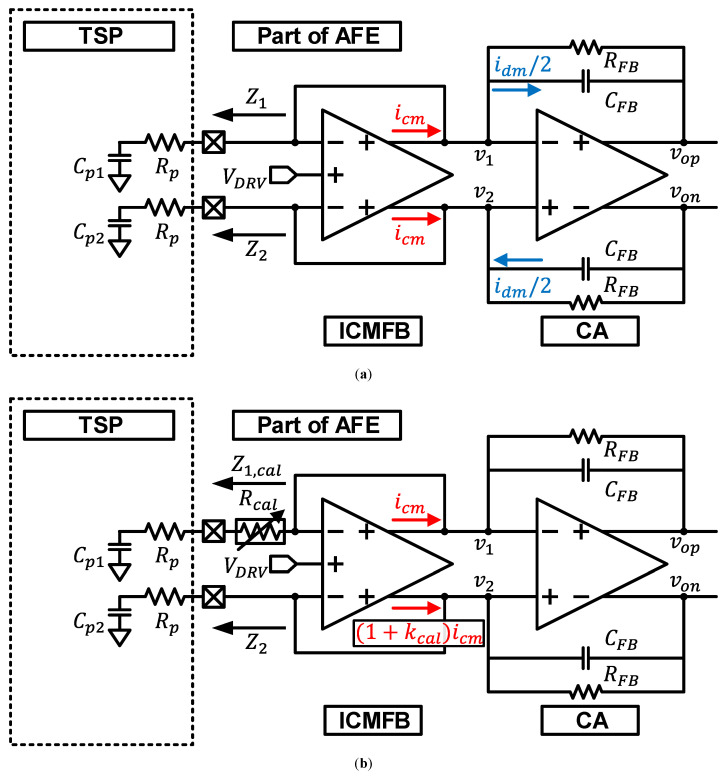
A fully-differential SCS system (**a**) without a self-capacitance mismatch calibration (**b**) with the proposed self-capacitance mismatch calibration technique, implemented with input resistance and driving strength control.

Figure 1a shows part of a fully-differential SCS system without the mismatch calibration technique. An input common-mode feedback amplifier (ICMFB) drives the self-capacitance of TSP, $C_{p1}$ and $C_{p2}$, and the parasitic resistance, $R_{p}$, by the driving signal, $V_{DRV}$ [6]. Note that ICMFB can only provide a common-mode current, $i_{cm}$. The feedback network of the AFE, which includes the ICMFB and charge amplifier (CA), tries to equalize the CA input voltages $v_{1}$ and $v_{2}$ in Figure 1a. When there is no self-capacitance mismatch, $i_{cm}$ from ICMFB is sufficient to match $v_{1}$ and $v_{2}$. Thus, $i_{dm}$, which is the differential-mode current generated from CA, and its corresponding $v_{out}$ offset is not generated, where $v_{out}=v_{op}-v_{on}$. However, if the self-capacitance mismatch exists, $i_{cm}$ alone cannot match $v_{1}$ and $v_{2}$. Therefore, $i_{dm}$ from CA is generated to equalize $v_{1}$ and $v_{2}$. Therefore, it becomes the output offset which degrades the dynamic range of the AFE. If we assume $C_{p2}=\left(1+m\right)C_{p1}$ and let $C_{p1}=C_{p}$, where *m* is self-capacitance mismatch ratio. Then, $i_{dm}$ and $v_{out}$ is obtained as

(1) $$i_{dm}=\frac{m}{1+\left(m/2\right)+s\left(1+m\right)R_{p}C_{p}}\;\cdot \;i_{cm}$$

(2) $$v_{out}=-\frac{R_{FB}}{1+sR_{FB}C_{FB}}\;\cdot \;i_{dm}$$

where $R_{FB}$ and $C_{FB}$ are resistor and capacitor in the CA feedback, respectively.

An approach to suppress $i_{dm}$ generation due to the self-capacitance mismatch can be considered, which adds the extra calibration current to the $v_{2}$ node in Figure 1a. With this approach, the required calibration current from the external amplifier, $i_{cal,ext}$, is expressed as

(3) $$i_{cal,ext}=\frac{m}{1+s\left(1+m\right)R_{p}C_{p}}\;\cdot \;i_{cm}.$$

Note that the denominator of Equation (3) contains the Laplace variable *s*, indicating that the expression involves a complex number. This implies that in order to completely remove the output offset, $i_{cal,ext}$ must be phase-shifted relative to $i_{cm}$. In other words, the system requires additional circuits to drive a phase-shifted signal from $V_{DRV}$, which is the self-capacitance driving signal. This leads to power and area inefficiency due to the generation of phase-shifted square or sinusoidal signals, as well as the requirement for an additional amplifier to drive the self-capacitance with a phase-shifted signal.

Our proposed solution is implementing programmable input resistance and ICMFB with driving strength control, as depicted in Figure 1b. By controlling $R_{cal}$ and $k_{cal}$, both the phase and magnitude of $v_{1}$ and $v_{2}$ can be matched without $i_{dm}$ generation from CA. Therefore, the $v_{out}$ offset is minimized.

## 3. Proposed Self-Capacitance Calibration

### 3.1. Ideal Calibration Condition

**Figure 2:**
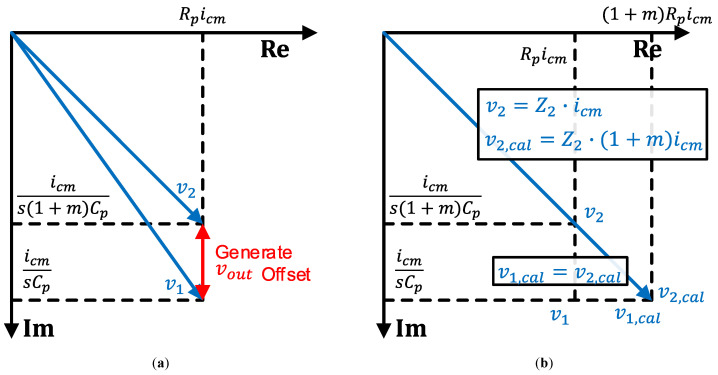
Phasor diagrams for $v_{1}$ and $v_{2}$, assuming the effect of the CA is excluded. (**a**) Before calibration, (**b**) after the proposed calibration, and ideal case when the offset is perfectly removed.

Figure 2a shows a phasor diagram for $v_{1}$ and $v_{2}$ before calibration, assuming that the effect of the CA, matching $v_{1}$ and $v_{2}$ by generating $i_{dm}$, is excluded. Because of the self-capacitance mismatch, the phasors of $v_{1}$ and $v_{2}$ have both magnitude and phase mismatches. To equalize this phasor mismatch, CA generates $i_{dm}$, which results in $v_{out}$ offset.

This $v_{out}$ offset can be removed by introducing the additional input resistance for $C_{p1}$ and increasing the driving strength of the ICMFB for $C_{p2}$, as depicted in Figure 2b. The phase mismatch can be removed by introducing additional input series resistance for $C_{p1}$ as, $R_{cal}=m\;\cdot \;R_{p}$. In other words, this increment of the input resistance equalizes the phase of two input impedance, $Z_{1,cal}$ and $Z_{2}$. Then, the magnitude mismatch between the phasors can be matched by increasing $C_{p2}$ driving strength of ICMFB, from $i_{cm}$ to $\left(1+m\right)i_{cm}$, resulting $Z_{1,cal}\;\cdot \;i_{cm}=Z_{2}\;\cdot \;\left(1+m\right)i_{cm}$. Since $v_{1,cal}=v_{2,cal}$, the $v_{out}$ offset is wholly removed after the proposed calibration.

### 3.2. Practical Calibration Process

**Figure 3:**
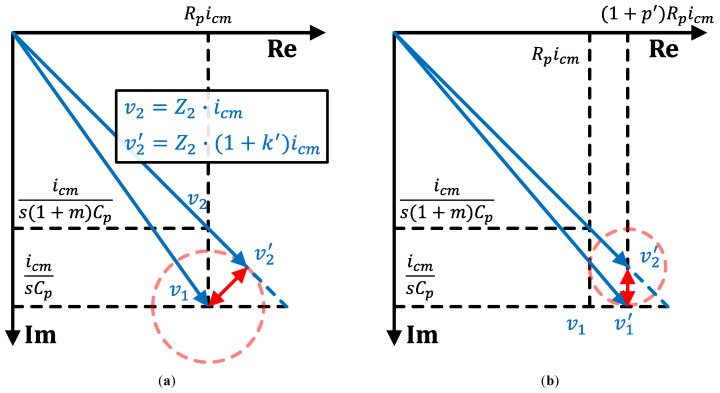
A single cycle of the proposed calibration consists of following two steps, explained with phasor diagrams of (**a**) driving strength adjustment and (**b**) input resistance adjustment.

In a practical usage scenario, the value of *m* is unknown for each TSP and its electrode channels. Additionally, the resolutions for programmable resistance and driver strength control are limited. Therefore, the implemented calibration requires an iterative process until the voltage phasor reaches the closest points to the ideal calibration point, $v_{1,cal}$ or $v_{2,cal}$. Figure 3 illustrates the steps of the iterative calibration process. First, the driving strength of ICMFB, which minimizes the output offset, is adjusted and found, as depicted in Figure 3a. In Figure 3a, $v_{2}$ moves to $v_{2}^{\prime }$ after the driving strength for $C_{p2}$ is increased from $i_{cm}$ to $\left(1+k^{\prime }\right)\;\cdot \;i_{cm}$. Since $v_{2}^{\prime }$ is the closest point to $v_{1}$, this is the first point where the driving strength adjustment minimizes the output offset of AFE. After that, the same action is performed for the input resistance control, as shown in Figure 3b. By increasing the input resistance for $C_{p1}$ from $R_{p}$ to $\left(1+p^{\prime }\right)\;\cdot \;R_{p}$, $v_{1}$ moves to $v_{1}^{\prime }$, which is the nearest point to $v_{2}^{\prime }$. Repeating these steps with multiple cycles makes the system gradually approach the ideal calibration point until the resolution of the driving strength and input resistance controls.

**Figure 4:**
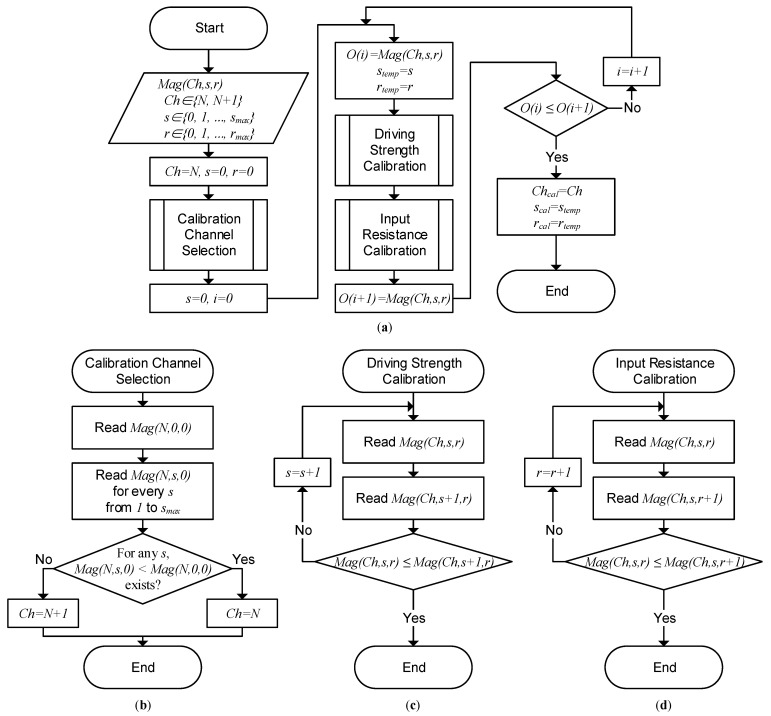
Flowchart of the proposed self-capacitance mismatch calibration. (**a**) The overall calibration process; (**b**) subroutine for calibration channel selection; (**c**) subroutine for driving strength calibration; and (**d**) subroutine for input resistance calibration.

Figure 4 shows a flowchart of the proposed self-capacitance mismatch calibration for a unit RX circuit sensing *N*th and N+1th RX (or TX) electrode channels. The proposed calibration process can be conducted by a micro controller unit (MCU). Mag(Ch,s,r) is the RX output magnitude when the calibration control signals are Ch, *s*, and *r*. Ch, *s*, and *r* are the calibration channel control, driving strength control and input resistance control, respectively. When Ch=N, the driving strength for the *N*th channel and the input resistance for the N+1th channel are increasing when the control codes *s* and *r* are increasing, respectively. For Ch=N+1, *s* controls the N+1th channel and *r* controls the *N*th channel.

For the first calibration run, control signals are initialized with Ch=N, s=0, and r=0. After the initialization, a subroutine for calibration channel selection, depicted in Figure 4b, is performed to decide Ch. The calibration channel selection subroutine checks the existence of any *s* that reduces the RX output magnitude while Ch=N. If such *s* exists, the calibration channel control is decided as Ch=N. If not, Ch=N+1. After the Ch decision, O(i), $s_{temp}$, and $r_{temp}$ are saved to memory. O(i) represents the RX output magnitude after performing the *i*th calibration iteration. During each iteration process, the driving strength calibration and input resistance calibration subroutines, as described in Figure 4c,d, respectively, are executed. During each iteration process, the driving strength calibration and input resistance calibration subroutines, as described in Figure 4c,d, respectively, are executed. Each subroutine sweeps and finds *s* and *r*, which minimizes Mag(Ch,s,r) in the given conditions. Note that, as a result of the driving strength calibration subroutine depicted in Figure 4c, Figure 3a illustrates the phasor diagram when the output mismatch is minimized. Similarly, Figure 3b is a result of the input resistance calibration subroutine shown in Figure 4d. With the newly found *s* and *r*, Mag(Ch,s,r) is saved to O(i+1) and then compared with the previously saved O(i). This iteration process is repeated until O(i)≤O(i+1), which means the system found the optimal $Ch_{cal}$, $s_{cal}$, and $r_{cal}$, which are closest to the ideal calibration condition.

### 3.3. Mathematical Analysis

We conducted a mathematical analysis to evaluate the degradation rate of $i_{dm}$ offset after each calibration cycle. After one cycle of the calibration, the phasors in Figure 2a, $v_{1}$ and $v_{2}$, are relocated as in Figure 3b, $v_{1}^{\prime }$ and $v_{2}^{\prime }$. In Figure 3b, $v_{1}^{\prime }$ and $v_{2}^{\prime }$ are located on the same X-coordinate and only differ their Y-coordinates, the same as $v_{1}$ and $v_{2}$ are in Figure 2a. Therefore, it is possible to interpret the X and Y coordinates of $v_{2}^{\prime }$ as $R_{p,1}i_{cm}$ and $i_{cm}/s\left(1+m_{1}\right)C_{p}$, respectively. Here, $m_{1}$ and $R_{p,1}$ represent the equivalent self-capacitance mismatch ratio and the equivalent parasitic resistance after one cycle of the calibration, respectively. With this perspective, it is possible to obtain the recurrence relation for $m_{1}$ and $R_{p,1}$, in terms of *m* and $R_{p}$. Moreover, the generalized recurrence relation for the equivalent self-capacitance mismatch ratio and the equivalent parasitic resistance after the *n*-cycle calibration, $m_{n}$ and $R_{p,n}$, respectively, are obtained as

(4) $$\left\{\begin{array}{cc} m_{n}=\left\{\frac{{\left(2\pi fR_{p,n-1}C_{p}\right)}^{2}\left(1+m_{n-1}\right)}{{\left(2\pi fR_{p,n-1}C_{p}\right)}^{2}\left(1+m_{n-1}\right)+1}\right\}\;\cdot \;m_{n-1} & \\ R_{p,n}=\left\{\frac{{\left(2\pi f\left(1+m_{n-1}\right)R_{p,n-1}C_{p}\right)}^{2}+\left(1+m_{n-1}\right)}{{\left(2\pi f\left(1+m_{n-1}\right)R_{p,n-1}C_{p}\right)}^{2}+1}\right\}\;\cdot \;R_{p,n-1} \end{array}\right.$$

with $m_{0}=m$ and $R_{p,0}=R_{p}$. Moreover, $i_{dm}$ after the *n*-cycle calibration, $i_{dm,n}$, is expressed as

(5) $$i_{dm,n}=\frac{m_{n}}{1+\left(m_{n}/2\right)+s\left(1+m_{n}\right)R_{p,n}C_{p}}\;\cdot \;i_{cm}$$

with $i_{dm,0}=i_{dm}$.

**Figure 5:**
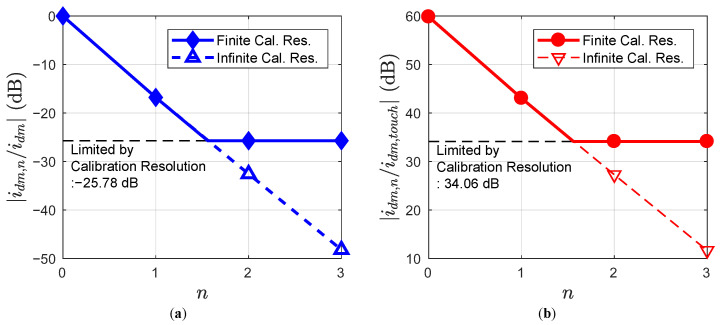
Degradation of output offset current over proposed calibration cycles (**a**) Compared to the output offset current before calibration (**b**) Compared to the output current induced by touch.

With (5), the $i_{dm,n}$ degradation over each calibration cycle can be evaluated numerically. Figure 5a shows the plot for $|i_{dm,n}/i_{dm}|$ after the *n*-th calibration cycle with f=150kHz, $R_{p}=1380\Omega$, $C_{p}=280\mathrm{pF}$, and m=0.12. Here, *f* represents the frequency of the touch signal. Note that for the analysis, $R_{p}$ includes not only the parasitic resistance of the TSP RX electrodes, but also the TSP-chip routing line resistance, and on-chip parasitic resistance. If we assume an infinite calibration resolution, $|i_{dm,n}/i_{dm}|$ continues to become smaller as the calibration cycle is repeated. However, in the practical design where the calibration resolution is finite, the offset removal performance of the proposed calibration is limited to −25.78dB after the second calibration cycle.

Figure 5b shows the ratio of the output offset current by the self-capacitance mismatch, $i_{dm,n}$, to the output current induced by touch, $i_{dm,touch}$. Note that the touch action on the TSP increases the self-capacitance of the nearby electrode. Thus, $i_{touch}$ can be obtained in the same way as (1) is obtained. Within 2 calibration cycles, the system reaches its calibration limit, and reduces $|i_{dm,n}/i_{dm,touch}|$ from 59.84 dB to 34.06 dB.

## 4. Implementation

**Figure 6:**
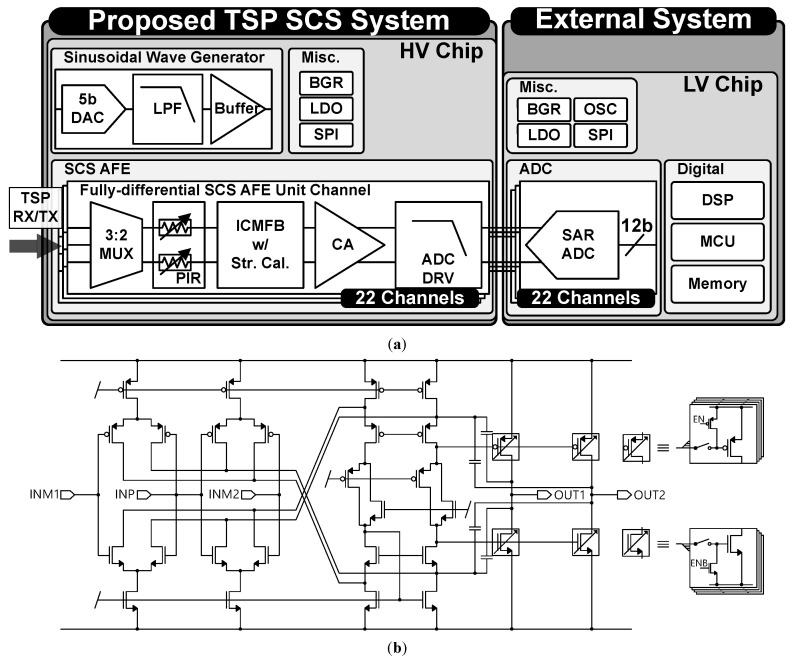
(**a**) Block diagram of the proposed TSP SCS system. (**b**) Schematic diagram of ICMFB with driving strength control.

Figure 6a illustrates the block diagram of the proposed TSP SCS system. The proposed work includes SCS AFE, sinusoidal wave generator, bandgap reference (BGR), low-dropout regulator (LDO), and serial peripheral interface (SPI) blocks in a high-voltage (HV) chip designed with a 130 nm CMOS process and 3V supply voltage. The proposed system is designed to work with an external low-voltage (LV) chip consisting of SAR ADC, digital blocks, and others. A 3:2 multiplexer (MUX) selects two adjacent TSP RX or TX electrodes to be sensed [6,9]. A sinusoidal wave generator generates a sinusoidal self-capacitance driving signal to minimize display image flickering due to the high voltage pulse wave driving [10,11]. The ICMFB drives the selected TSP electrodes pair with a 150 kHz and 2.8 V_pp_ sinusoidal wave. A CA amplifies the input current difference due to the touch, and an ADC driver (ADC DRV) drives the input of SAR ADC in the external LV chip while providing anti-aliasing filtering. The LV chip samples AFE output, processes touch data using DSP, and controls both HV and LV chips with MCU.

For the proposed self-capacitance mismatch calibration, a programmable input resistor (PIR) block and the ICMFB with a driving strength control feature are implemented. The PIR, which consists of a poly resistor and switch arrays, is implemented to introduce input series resistance difference between the fully-differential pairs. Due to the trade-off between the area consumption and the calibration resolution, the PIR is designed as a 3-bit control with 20 Ω step. Figure 6b shows the structure of ICMFB with driving strength control. The 4-bit binary driving strength control code selects the number of the output stage CMOS that should be turned on for OUT1 and OUT2 separately. Therefore, the ICMFB can introduce a driving strength difference up to 38.75%, with 1.25% LSB.

## 5. Simulation Results

**Figure 7:**
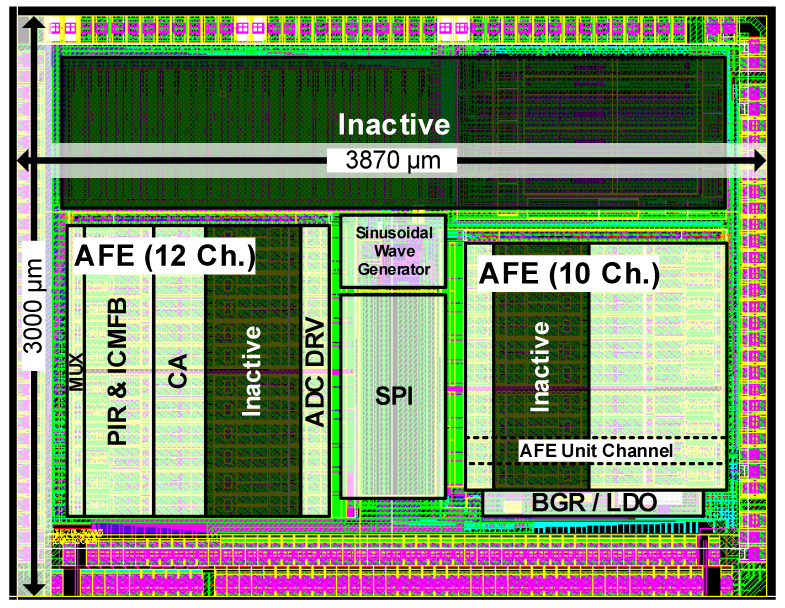
Layout of the proposed TSP SCS system.

The proposed TSP SCS system is designed with a 130 nm CMOS process. Figure 7 shows the layout of the proposed TSP SCS system. The active area of the proposed TSP SCS system is 3.15 mm${}^{2}$.

**Figure 8:**
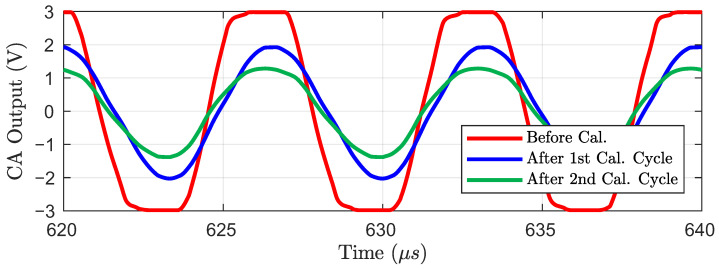
CA output waveform with and without the proposed calibration.

Figure 8 shows the CA output before and after the proposed calibration. The driving signal is a 150 kHz and 2.8 V_pp_ sinusoidal wave. An OLED TSP model with $C_{p}=280\mathrm{pF}$ and $R_{p}=1380\Omega$ was used for simulation. To assume the worst case, a 12% self-capacitance mismatch between RX electrodes was applied, which is equivalent to 33.6 pF mismatch. Therefore, the CA differential output was saturated before applying the proposed calibration. After the first cycle of the proposed calibration, the self-capacitance mismatch no longer caused saturation in the CA output. Thus, the self-capacitance variation by touch, which is 40 fF for our design target TSP model, is detectable by AFE. As the proposed calibration cycle is repeated, the output offset decreases. In other words, the headroom for the increased gain of AFE is acquired and the SNR of the SCS system is increased with the repetition of the proposed calibration cycles. Due to the limited calibration resolution and the large signal non-linearity of the ICMFB, the proposed calibration reached its maximum output offset removal performance after the two calibration cycles were performed.

**Figure 9:**
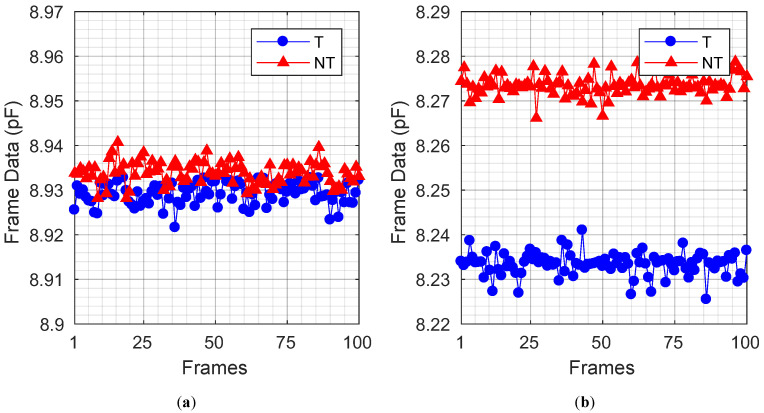
Frame data plots, with 12% self-capacitance mismatch (**a**) before calibration (**b**) after calibration.

Figure 9 shows frame data plots before and after the proposed calibration. Each frame data was obtained by applying digital signal processing, which includes down-conversion, cascaded integrator-comb (CIC) filtering, and integration to each 2 ms time-domain data, resulting in a single integer value, called frame data. Therefore, the frame data represents the received touch signal intensity sensed over a 2 ms period. Note that, in Figure 9, the unit of the frame data is converted to pF by correlating the difference between the average touch (T) and non-touch (NT) frame data to the known $\Delta C_{p}$ value, which is provided by the TSP manufacturer. To obtain the SNR with a 120 Hz frame rate, the duration of each time-domain data is 2 ms. The time-domain data include the circuit noise of the proposed TSP SCS system, as well as modeled external noise, such as display noise. The external noise was modeled using a method based on [12]. Because of the saturation of the AFE, the TSP SCS system cannot distinguish touch (T) and non-touch (NT) state frame data, as shown in Figure 9a. After the proposed calibration resolves the saturation, the T and NT frame data are now distributed separately without overlapping, making them distinguishable. Therefore, touch distinction becomes possible after the proposed calibration, as shown in Figure 9b.

Table 1 compares the SNR of the proposed TSP SCS system before and after the proposed calibration is performed, in the presence of a 12% self-capacitance mismatch with a 120 Hz frame rate. SNR, $TouchStrength_{Sensed}$, and $Noise_{RMS}$ were calculated from the frame data in Figure 9, as follows: [13,14]

(6) $$SNR\left(dB\right)=20\log_{10}(|TouchStrength_{Sensed}/Noise_{RMS}\left|\right)$$

(7) $$TouchStrength_{Sensed}=AVG_{T,100}-AVG_{NT,100}$$

(8) $$Noise_{RMS}=\sqrt{\frac{\sum_{n=1}^{100}{\left(T\left[n\right]-AVG_{T,100}\right)}^{2}}{100}}$$

where T[n], $AVG_{T,100}$, and $AVG_{NT,100}$ are the *n*-th frame data when touched, the average of 100 frame data when touched, and the average of 100 frame data when not touched, respectively. $TouchStrength_{Sensed}$ is defined as the difference between $AVG_{T,100}$ and $AVG_{NT,100}$, and it represents the strength of touch signal variation caused by changes in $\Delta C_{p}$ due to touch. $Noise_{RMS}$ is the root-mean-square value of the 100 frame data when touched, with $AVG_{T,100}$ as a baseline. Then, SNR is evaluated by dividing $TouchStrength_{Sensed}$ by $Noise_{RMS}$. The proposed calibration resolves the saturation of the AFE stage and provides headroom for higher AFE gain, thereby increasing the SNR by 19.54 dB.

**Figure 10:**
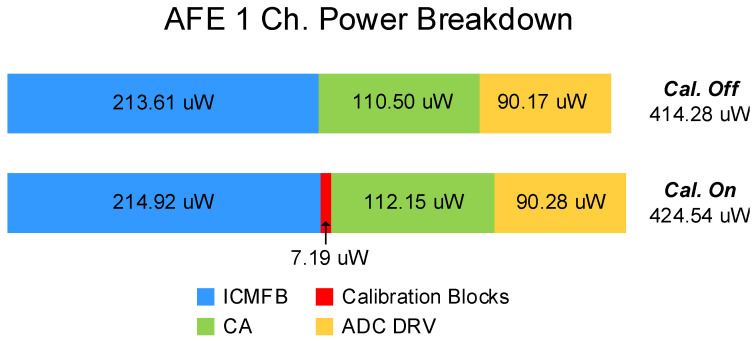
Power breakdown of a single AFE channel, comparing before and after the calibration is performed.

Figure 10 illustrates the power breakdown for a single AFE channel, comparing before and after the calibration is performed. In the “Cal.Off” state, the proposed calibration is turned off, and the self-capacitance mismatch is not modeled. As a result, the output offset is not generated in the “Cal. Off” state. In the “Cal.On” state, the proposed calibration has been performed, minimizing the output offset while exposed to 12% self-capacitance mismatch. The power breakdown shows that only an additional 10.26 μW is consumed after the proposed calibration is performed. Furthermore, the calibration blocks, including PIR and the switched output devices of ICMFB, consume 7.19 μW, which accounts for 1.69% of the total power consumption in the “Cal. On” state. Additionally, the calibration block only takes 6.31% of the active area in a single-channel AFE. These results demonstrate that the proposed calibration can be implemented with minimized power and area consumption, making it an efficient solution for addressing self-capacitance mismatches.

**Figure 11:**
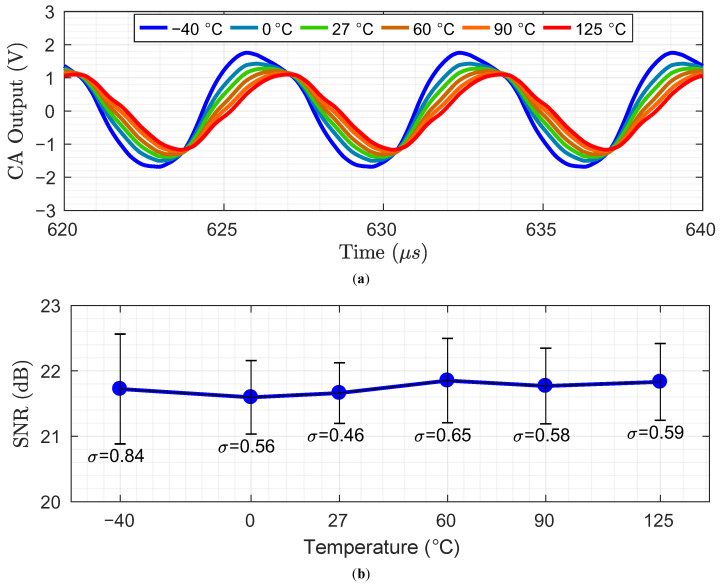
Performance consistency under temperature variation with optimal calibration setting found at 27 ${}^{\circ }C$, while exposed to a 12% self-capacitance mismatch (**a**) CA output waveform (**b**) SNR results.

Figure 11 demonstrates the performance consistency of the system under temperature variation with optimal calibration setting found at 27 ${}^{\circ }C$, while exposed to a 12% self-capacitance mismatch. Figure 11a shows the CA output waveform variation. With the optimal calibration setting found at 27 ${}^{\circ }C$, the CA output offset amplitude varies inversely with temperature changes. However, this temperature induced variation in the CA output offset does not cause saturation of the CA output, ensuring the proposed system’s SNR remains stable, as shown in Figure 11b. For Figure 11b, 10 SNR results are obtained at each temperature points, and their average is plotted. The error bars indicate the standard deviation of these results. This SNR result demonstrates that the system performance remains stable under temperature variation, even without updating the calibration setting for temperature change. Based on the demonstrated stable SNR performance under temperature variation without updating the calibration setting, the proposed calibration does not require adjustments for temperature variation.

Table 2 is the performance summary of the proposed TSP SCS system and the previous works. The proposed work achieves comparable 40.98 dB SNR with a 120 Hz frame rate while consuming 6.2 mW. Even under the worst self-capacitance mismatch case, the proposed SCS system was able to sense the self-capacitance variation by touch with 20.89 dB SNR after the proposed self-capacitance mismatch calibration was performed. Note that the fair comparison of SNR, power consumption, or FoM [15] is difficult because they heavily depend on TSP electrical characteristics [16].

## 6. Conclusions

This paper presents a self-capacitance mismatch calibration technique that reduces the dynamic range degradation of fully-differential AFE for TSP SCS. By introducing input resistance and driving strength differences between the fully-differential input pair, the output offset due to the self-capacitance mismatch is minimized without excessive power and chip area consumption. With the proposed calibration technique, the SNR for the TSP SCS system is maximized or recovered even in the situation when the large self-capacitance mismatch saturates the AFE.

## Figures and Tables

**Table 1:** SNR comparison table with 12% self-capacitance mismatch.

	*TouchStrength_Sensed_*	*Noise_RMS_*	*SNR*
Before calibration	4.64fF	3.97fF	1.35
After calibration	40 fF	3.61fF	20.89

**Table 2:** Performance comparison with reported TSP self-capacitance sensing systems.

	Proposed ${}^{\left(a\right)}$	[5]	[6]	[7]	[8]
Process	130nm CMOS	130nm CMOS	180nm CMOS	180nm CMOS	180nm BCD
TSP Size	6.87-inch	1.3-inch	5-inch	5.8-inch	10.1-inch
TSP # of Electrodes	TX: 18 RX: 40	SC: 16	TX: 28 RX: 16	TX: 16 RX: 33	TX: 55 RX: 34
Frame Rate	120 Hz	330 Hz	120 Hz	120 Hz	240 Hz
Power	6.2 mW	1.04 mW	10.2 mW	2.1 mW	16.3 mW
$C_{p}$ Offset Calibration	Yes	Yes	No	Yes	Yes
SNR	40.98 dB	47.2 dB	53 dB	32 dB	39 dB
SNRw/$C_{p,mis}$	20.89 dB ${}^{\left(b\right)}$	N/A	N/A	N/A	N/A
Chip area	3.15 m m ${}^{2}$	0.128 m m ${}^{2}$	N/A	0.12 m m ${}^{2}$	10.22 m m ${}^{2}$

^(*a*)^ Based on layout post-extraction simulation. ^(*b*)^ Under 12% self-capacitance mismatch (C_p,mis_ = 33.6 pF.

## Data Availability

Not applicable.
